# New organizational principles and 3D cytoarchitectonic maps of the dorsolateral prefrontal cortex in the human brain

**DOI:** 10.3389/fnimg.2024.1339244

**Published:** 2024-02-22

**Authors:** Ariane Bruno, Kimberley Lothmann, Sebastian Bludau, Hartmut Mohlberg, Katrin Amunts

**Affiliations:** ^1^Institute of Neuroscience and Medicine (INM-1), Research Centre Jülich, Jülich, Germany; ^2^Cécile and Oskar Vogt Institute for Brain Research, Medical Faculty and University Hospital Düsseldorf, Heinrich Heine University Düsseldorf, Düsseldorf, Germany

**Keywords:** 3D brain mapping, Julich-Brain, interindividual variability, probability maps, Human Brain Atlas

## Abstract

Areas of the dorsolateral prefrontal cortex (DLPFC) are part of the frontoparietal control, default mode, salience, and ventral attention networks. The DLPFC is involved in executive functions, like working memory, value encoding, attention, decision-making, and behavioral control. This functional heterogeneity is not reflected in existing neuroanatomical maps. For example, previous cytoarchitectonic studies have divided the DLPFC into two or four areas. Macroanatomical parcellations of this region rely on gyri and sulci, which are not congruent with cytoarchitectonic parcellations. Therefore, this study aimed to provide a microstructural analysis of the human DLPFC and 3D maps of cytoarchitectonic areas to help address the observed functional variability in studies of the DLPFC. We analyzed ten human post-mortem brains in serial cell-body stained brain sections and mapped areal boundaries using a statistical image analysis approach. Five new areas (i.e., SFG2, SFG3, SFG4, MFG4, and MFG5) were identified on the superior and middle frontal gyrus, i.e., regions corresponding to parts of Brodmann areas 9 and 46. Gray level index profiles were used to determine interregional cytoarchitectural differences. The five new areas were reconstructed in 3D, and probability maps were generated in commonly used reference spaces, considering the variability of areas in stereotaxic space. Hierarchical cluster analysis revealed a high degree of similarity within the identified DLPFC areas while neighboring areas (frontal pole, Broca's region, area 8, and motoric areas) were separable. Comparisons with functional imaging studies revealed specific functional profiles of the DLPFC areas. Our results indicate that the new areas do not follow a simple organizational gradient assumption in the DLPFC. Instead, they are more similar to those of the ventrolateral prefrontal cortex (Broca's areas 44, 45) and frontopolar areas (Fp1, Fp2) than to the more posterior areas. Within the DLPFC, the cytoarchitectonic similarities between areas do not seem to follow a simple anterior-to-posterior gradient either, but cluster along other principles. The new maps are part of the publicly available Julich Brain Atlas and provide a microstructural reference for existing and future imaging studies. Thus, our study represents a further step toward deciphering the structural-functional organization of the human prefrontal cortex.

## 1 Introduction

The DLPFC plays diverse roles in the performance of executive functions, such as attention (Rowe and Passingham, 2001; Tanji and Hoshi, 2008), abstract reasoning (Bernardi et al., 2020), response inhibition (Blasi et al., 2006), planning (Crescentini et al., 2012), cognitive flexibility (Badre and Nee, 2018; Badre et al., 2021), behavior (Shallice and Burgess, 1991; Barraclough et al., 2004), and working memory (Rowe and Passingham, 2001; Petrides, 2005; Warden and Miller, 2010; Barbey et al., 2013). There is evidence that the DLPFC does not seem to function as a single unit but can be functionally differentiated along an anterior-posterior and dorsal-ventral axis (O'Reilly, 2010; Goulas et al., 2012; Cieslik et al., 2013; Sallet et al., 2013; Badre and Nee, 2018). For example, Jung et al. (2022) divided the DLPFC along the rostrocaudal and dorsoventral axes into seven distinct areas based on different structural and functional connectivity patterns.

**Figure 1:**
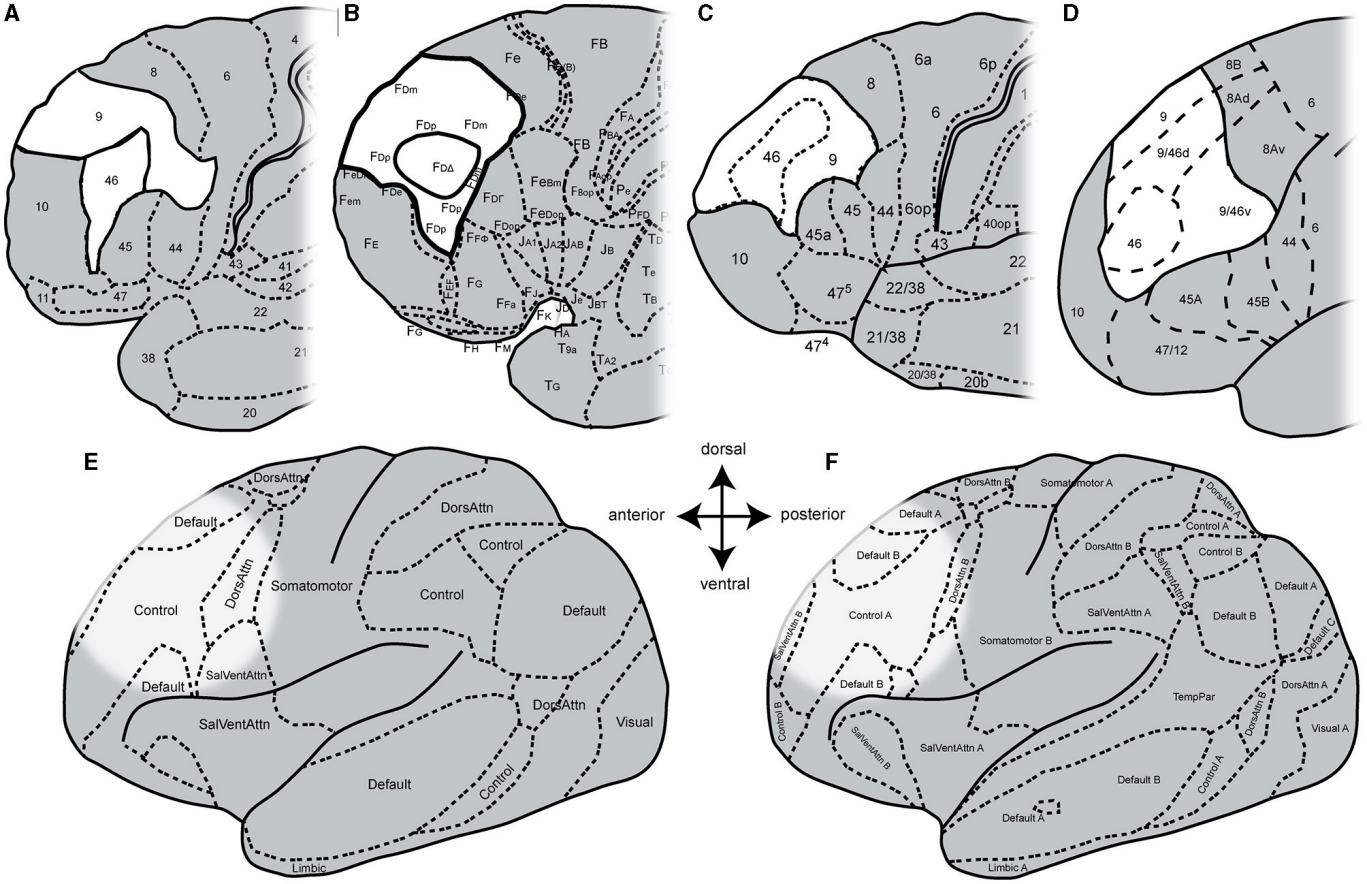
Schematic maps of the human prefrontal cortex. Adapted cytoarchitectonic maps by **(A)** Brodmann (1909), **(B)** von Economo and Koskinas (1925), **(C)** Sarkissov et al. (1955), and **(D)** Petrides and Pandya (1999). Adapted functional maps with seven networks **(E)** and 17 networks **(F)** by Yeo et al. (2011). Areas of the DLPFC are highlighted in white and bold black lines (in cytoarchitectonic maps). The DLPFC in the functional maps is highlighted due to histological location.

To date, the functional heterogeneity of the DLPFC has been reflected in existing anatomical maps that rely on either macroanatomy (Desikan et al., 2006; Destrieux et al., 2010; Klein and Tourville, 2012) or cytoarchitectonic features (Brodmann, 1909; von Economo and Koskinas, 1925; Sarkissov et al., 1955; Rajkowska and Goldman-Rakic, 1995a; Petrides and Pandya, 1999) (Figure 1). Brodmann created the first histological brain map with a parcellation of the human cortex into 43 areas (Brodmann, 1909). He divided the human DLPFC into two areas: BA9, located on the superior frontal gyrus (*sfg*) and caudal parts of the middle frontal gyrus (*mfg*), and BA46, which can be found on the remaining portion of the *mfg* and inferior frontal gyrus (*ifg*) with a ventral border to BA45 and a rostral border to the frontal pole area BA10 (Figure 1A). Subsequent microstructural parcellations of the DLPFC differ in the neighborhood relationship of prefrontal areas and volume ratio of these two areas (von Economo and Koskinas, 1925; Sarkissov et al., 1955; Rajkowska and Goldman-Rakic, 1995a; Petrides and Pandya, 1999). Whereas, Brodmann's BA46 was shown as an area extending into BA9, the following map depicted BA46 as an island surrounded by BA9 (von Economo and Koskinas, 1925) (Figure 1B). Rajkowska and Goldman-Rakic delineated the DLPFC into more than two distinct microstructural areas (Rajkowska and Goldman-Rakic, 1995a). They defined so-called transitional areas 9–46 with cytoarchitectonic features of both regions 9 and 46. Petrides and Pandya further subdivided the transition areas into a dorsal 9/46 (9/46d) and a ventral part 9/46 (9/46v) with an embedded area 46 (Figure 1D) by comparison with macaque brains (Petrides and Pandya, 1999).

In addition to these cytoarchitectonic maps from post-mortem brains, several functional and structural maps were created based on *in vivo* imaging (Glasser et al., 2016; Gordon et al., 2016; Schaefer et al., 2018; Doucet et al., 2019; Dadi et al., 2020) relying, for example, on connectivity (Fan et al., 2016; Jung et al., 2022) or fMRI (Yeo et al., 2011; Shen et al., 2013). Moreover, dictionaries of functional modes (DiFuMo) have been created to provide finely resolved atlases of functional modes (Dadi et al., 2020) based on millions of fMRI functional brain volumes, such as resting-state fMRI (Yeo et al., 2011) to create a reference functional brain parcellation. Here, the parcellation of the cortex depends on the number of networks (Figures 1E, F). However, the existing functional parcellations of the cortex cannot be fully aligned with the existing microstructural maps. This may also be due to the high variability in the size and location of fMRI activations found across the different studies (Nee et al., 2007; Kohn et al., 2014) as well as the complex macroanatomical structure of the frontal lobe, to name only a few of many factors.

At the macroscopic level, refined parcellations of the human cortex from MRI-based studies (Goulas et al., 2012; Cieslik et al., 2013; Sallet et al., 2013; Glasser et al., 2016; Donahue et al., 2018) were published. However, segmentation of human DLPFC is challenging due to the interindividual variation in sulcus patterns (Vogt and Vogt, 1926; Ono et al., 1990; Huttner, 2004; Miller et al., 2021; Bruno et al., 2022; Willbrand et al., 2023). At the microscopic level, there is a lack of information in three-dimensional (3D) space, which does not allow a direct superimposition of previous maps with datasets from functional imaging studies (Zilles and Amunts, 2010). To address these problems, generating probabilistic cytoarchitectonic maps in known reference spaces presents a suitable approach. Thus, the issue of interindividual tertiary sulci (Miller et al., 2021; Willbrand et al., 2023) is addressed by probabilistic maps, as well as the microstructural challenges are clarified by cytoarchitectonic analyses.

A recent study of our group identified four cytoarchitectonically distinct areas within the human DLPFC in the superior frontal sulcus (*sfs*) and the *mfg* using a reliable reproducible cytoarchitectonic mapping approach (Amunts et al., 2020). It demonstrated that the DLPFC is more grained than previously assumed (Bruno et al., 2022), i.e., that the human DLPFC is cytoarchitectonically composed of more regions than previously assumed (Brodmann, 1909; von Economo and Koskinas, 1925; Rajkowska and Goldman-Rakic, 1995a; Petrides and Pandya, 1999).

Starting from this work, the present study aimed to analyze remaining parts of the DLPFC to understand the DLPFC better and provide a microstructural reference for functional studies. These mainly include regions on the *sfg* and *mfg* as well as the adjoining sulci bordering already delineated areas such as frontal pole area Fp1 (Bludau et al., 2014), the premotor area 6d3 (Sigl, 2018), subdivision of area 8 (8d1, 8d2, 8v1, and 8v2) (Amunts et al., 2021), anterior DLPFC areas (SFS1, SFS2, MFG1, and MFG2) (Bruno et al., 2022), areas of the Broca region (44 and 45) of the ventral lateral prefrontal cortex (VLPFC) (Amunts et al., 1999), and areas ifs1 and ifs3 in the inferior frontal sulcus (*ifs*) (Ruland et al., 2022). To address the interindividual variability of the DLPFC, probabilistic maps will be generated for each identified area in two common stereotaxic spaces (Amunts et al., 2020) to provide a reference system for future complementary functional and multimodal approaches. Area volumes and cell body fractions will be calculated and analyzed for interhemispheric and sex differences.

## 2 Materials and methods

### 2.1 Histological processing of post-mortem brains

Ten human post-mortem brains (five male, mean age 67, range of age 30–85 years) were used for cytoarchitectonical analysis. The brains were obtained from the body donor program of the Department of Anatomy at the University Hospital Düsseldorf of the Heinrich-Heine-University. Ethical approval and documented written informed consent were obtained in accordance with legal requirements (Faculty of Medicine, Heinrich-Heine-University, Düsseldorf, Germany, ethics approval number 4863). The clinical history did not reveal any psychiatric or neurological disease. The histological procedure and subsequent image analysis were performed as described in detail (Amunts et al., 2020). The post-mortem delay was between 12 and 24 hours. After removal of the brain from the skull, the brains were fixed in formalin or Bodian's fixative for approximately 3–6 months, embedded in paraffin and sectioned in 6,000–7,500 coronal slices using a large-scale microtome (20 μm thick serial whole-brain sections). Every 15th section was stained for cell bodies with a modified silver staining method (Merker, 1983). After digitization, every 30th to 60th section (distance between sections: 0.6 and 1.2 mm) was used for observer-independent cytoarchitectonical analysis.

### 2.2 Observer-independent identification of cytoarchitectonic borders based on the gray level index (GLI)

**Figure 2:**
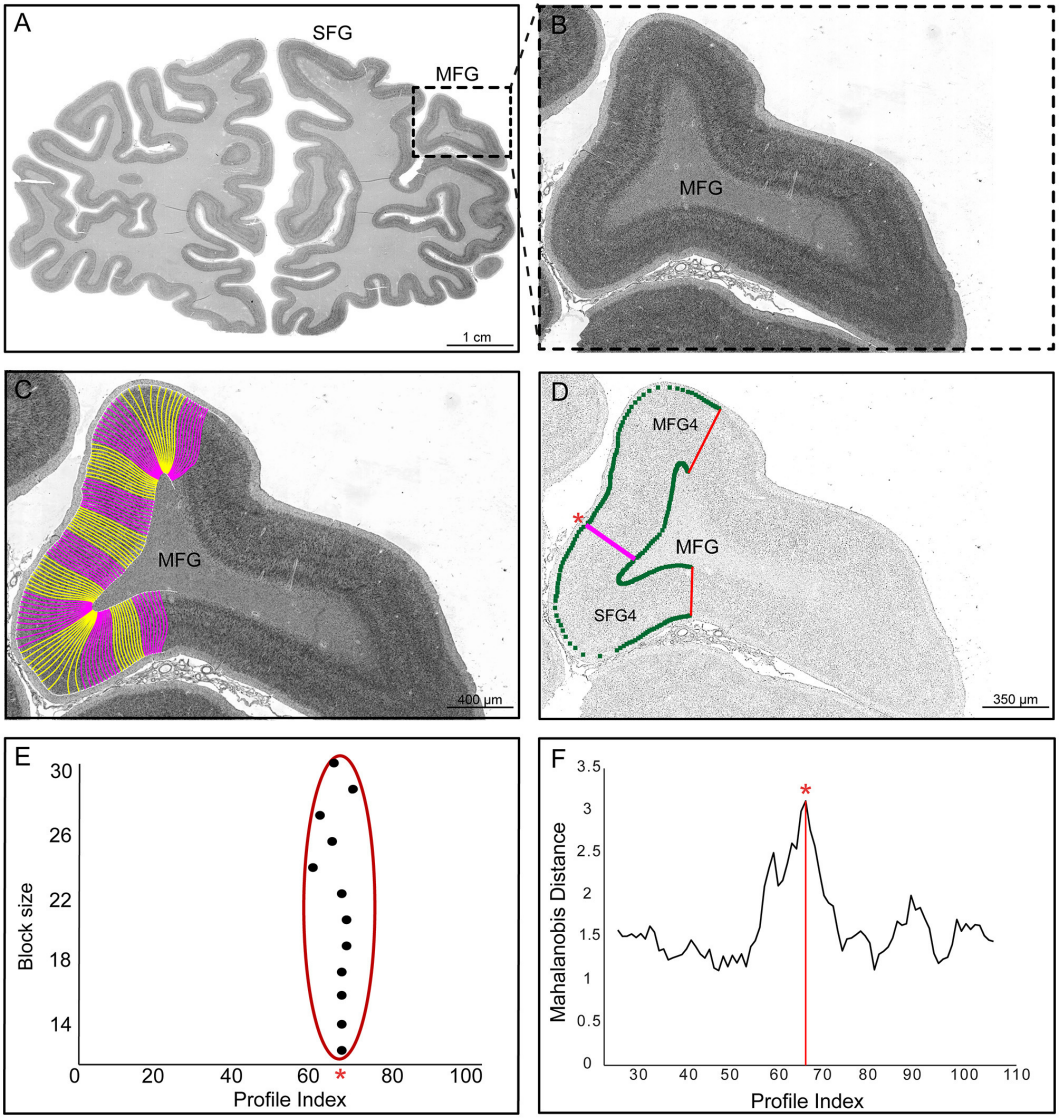
User-independent mapping approach. **(A)** The cell body stained coronal section (scale: 1 cm) from one of the ten brains with the region of interest (ROI, box). **(B)** The ROI (box) defines the examined cytoarchitectonic area on the middle frontal gyrus (MFG). **(C)** The ROI shows outer and inner contour lines with traverses (pink and yellow) (scale: 400 μm). **(D)** The digitized ROI was converted into gray level index (GLI) images via a Matlab-based script. Inverted GLI image of the ROI is shown with a statistically significant border (pink line) between areas SFG4 and MFG4 (scale: 350 μm). **(E)** Positions of significant maxima (see asterisk) along the profiles for different block sizes. **(F)** The significant maximum at profile number 67 is marked with an asterisk in the Mahalanobis distance function for one block size.

Borders were determined using observer-independent image analysis and statistical tools designed to identify significant changes in the laminar pattern, i.e., the cytoarchitecture (Schleicher et al., 1999, 2009; Amunts et al., 2020) (Figure 2). Images with a resolution of 1 μm/pixel (~8 Gb per image, 8 bit) were obtained from histological sections of 10 human post-mortem brains (Table 1). A high-throughput brightfield microscope (TissueScope LE120, Huron Digital Pathology) was used to scan the sections on which the regions of interest (ROI), including the superior and middle frontal gyrus, were analyzed (Figure 2A).

**Table 1:** Post-mortem brains used for the cytoarchitectonic analysis of the DLPFC, including the BigBrain B20 (Amunts et al., 2013).

**Brain no**.	**Sex**	**Age [years]**	**Cause of death**	**Fresh brain weight [g]**
B01	Female	79	Bladder carcinoma	1.350
B04	Male	75	Acute glomerulonephritis	1.349
B05	Female	59	Cardiorespiratory insufficiency	1.142
B06	Male	54	Myocardial infarction	1.622
B08	Female	72	Cardiorespiratory insufficiency	1.216
B09	Female	79	Cardiorespiratory insufficiency	1.110
B10	Female	85	Myocardial infarction	1.046
B11	Male	74	Myocardial infarction	1.381
B20	Male	65	Cardiorespiratory insufficiency	1.392
B21	Male	30	Bronchopneumonia	1.409

A Matlab-based script (The MathWorks, Inc., Natick, MA, USA) was used to convert the digitized ROIs into gray level index (GLI) images (Schleicher et al., 2009) (Figure 2B). The GLI represents the cytoarchitectonic organization (Schleicher et al., 1986) by estimating cell body volume fraction (Wree et al., 1982) in a 16 × 16 μm^2^ measuring field. Equidistant traverses were then calculated between the contour lines marking the layer I/II boundary (outer contour line) and the layer VI/white matter boundary (inner contour line) using a physical model of electric field trajectories (Jones et al., 2000). The outer and inner lines were interactively determined in each GLI image using Matlab scripts in-house written. GLI profiles were extracted perpendicular to the cortical layers (Figure 2C). These GLI profiles represent the changes of the GLI from the cortical surface to the white matter and characterize the laminar changes in the cytoarchitecture of the ROI (Schleicher et al., 2009). Each profile was normalized to a cortical depth of 100% in order to compare cortices of different thicknesses. The GLI profiles were characterized by a 10-dimensional feature vector consisting of mean GLI, cortical centroid depth, standard deviation, skewness, kurtosis, and similar parameters for the first derivatives (Schleicher et al., 2009). The advantage of the GLI method is that it is based on the cell packing density and thus minimizes the influence of the color intensities of the brain sections. Although nuclei of glial cells and endothelial cells are also stained, their area-specific areal-specific or laminar differences do not seem to influence the present analysis and parcellation (Wree et al., 1982).

The Mahalanobis distance, a multivariate distance measure, was used to quantify differences between feature vectors of adjacent blocks of GLI profiles (Mahalanobis et al., 1949; Schleicher et al., 1999) (Figures 2D, E). The shapes of adjacent profiles reflected by the ten-dimensional feature vector were compared for observer-independent border detection (Schleicher and Zilles, 1990; Schleicher et al., 2005). Each profile block consisted of 12 to 30 profiles. The distance for each localization along the cortical band was calculated using a sliding window procedure. Maximum Mahalanobis distances indicated areal boundaries (Figure 2F). Hotelling's T2 test with Bonferroni correction for multiple comparisons (*p* < 0.001) was performed to determine the significance of the boundaries. Each border was verified by visually inspecting the histological images to ensure histological and cutting qualities. Borders were accepted if consistently detected in the same positions across multiple block sizes and at least three adjacent sections.

### 2.3 Hierarchical clustering of mean areal GLI profiles

Hierarchical cluster analysis was performed to detect similarities between the new DLPFC areas and the neighboring frontal pole areas (Fp1, Fp2) (Bludau et al., 2014), anterior DLPFC areas (Bruno et al., 2022), areas 44 and 45 of Broca's region (Amunts et al., 1999), subdivisions of areas 8 [8d1, 8d2, 8v1, and 8v2 (Amunts et al., 2021)] and areas of the motoric region, like 6d1, 6d2, and 6d3 (Amunts et al., 2021), and 4a, and 4p (Geyer et al., 1996). Three sections with an average of 15–20 profiles per hemisphere were extracted for each area and brain, i.e., approximately 45 profiles per hemisphere (900 profiles total). Sections were selected that were free of histological artifacts, large vessels and that were not cut tangentially. Feature vectors were generated for each area, and discriminant analyses were calculated using Euclidean distance and the Ward-linking method (Ward, 1963) based on the mean GLI profiles (Schleicher et al., 1999). A high Euclidean distance indicates high cytoarchitectural difference and low structural similarity, and vice versa. The results were visualized using a dendrogram.

### 2.4 Reconstruction of cortical areas and stereotaxic maps

The area extent of both hemispheres was completely labeled in digitized high-resolution histological sections (1,200 dpi; ~20 μm/pixel; 8-bit gray value resolution) by the in-house software “Section Tracer Online Tool”. The same deformation fields calculated for the histological volumes of the 10 post-mortem brains (Amunts et al., 2020) were then used to reconstruct the cytoarchitectonic regions in 3D.

The brain's 3D reconstruction was created using the structural magnetic resonance image (MRI) 3D data set of the fixed brains before sectioning and high-resolution flatbed scans of stained histological sections (Amunts et al., 1999). The datasets were compared, and adjustments were made to account for any deformations or shrinkage (Henn et al., 1997; Amunts et al., 2004; Bludau et al., 2014). To create a stereotaxic map and account for anatomical variability, the delineated areas of all examined brains were normalized and transferred to Montreal Neurological Institute (MNI) Colin27 and the non-linear asymmetric MNI152 2009c (ICBM152casym) template using linear and non-linear elastic registration tools (Evans et al., 1992; Henn et al., 1997; Hömke, 2006). The reference brain templates were used to superimpose the new cytoarchitectonic areas and generate probabilistic maps (Amunts et al., 2020).

These maps illustrate the intersubject variability of a cortical area at a given position in the reference brain. Probabilities were color-coded from dark blue (high variability) to red (low variability). These maps provide a metric of intersubject variability: the higher the probability, the lower the cerebral intersubject variation in that cortical area. These probability maps were used to create a continuous, non-overlapping maximum probability map (MPM) of the newly identified and previously mapped areas, assigning each voxel to the area with the highest probability corresponding to that particular voxel (Eickhoff et al., 2005). Centers of gravity were calculated. All area maps are publicly available via the Julich Brain Cytoarchitectonic Atlas (Amunts et al., 2020) and the HBP Human Brain Atlas as part of EBRAINS (https://ebrains.eu/service/human-brain-atlas).

### 2.5 Volumetric analysis

Interhemispheric and sex differences in the volumes of the new areas were analyzed and compared between brains. An in-house software labeled cortical areas in histological, high-resolution (7,000 × 6,000 pixels), and cortical sections (1.2 mm apart, every 60th section). The volumes of the ROIs were calculated using the following formula:


$$\begin{array}{l} V=s\;\cdot \;T\;\cdot \;x\;\cdot y\;\cdot \;\sum N_{i}\;\cdot F\; \end{array}$$

*V* is the volume of the ROI (mm^2^), *s* the distance between the examined sections (number of sections), *T* is the thickness of a histological section (20 μm), *x* is the width of a pixel (0.02116 mm), y is the height of a pixel (0.02116 mm), Σ*N*_*i*_ is the sum of the areas of the structure overall sections (in pixels), and *F* is the shrinkage factor of each brain. To compare the volumes of DLPFC areas from brains of different volumes, they were individually corrected for shrinkage due to histological processing (Amunts et al., 2007).

Normalized volumes were analyzed using mixed model repeated measures ANOVA (within factor, area and hemisphere; between factor, sex) to test for volume significance. Normality was tested by Shapiro–Wilk and sphericity by Mauchly. Levene's test was used to assess the homogeneity of error variances. A significance level of α = 0.05 was used for all tests.

### 2.6 Analysis of cytoarchitectonic patterns

GLI profiles characterize the changes in the volume fraction of cell bodies (Wree et al., 1982), i.e., the laminar pattern of the areas. An increased volume fraction of cell bodies corresponds to a reduced proportion of neuropil, i.e., less axonal, dendritic, and synaptic space. Mean GLI values were calculated based on 15–20 profiles in three histological sections per area, hemisphere, and brain. Mean GLI values were studied for sex, inter-hemispheric and inter-area differences. Statistical analyses were performed using mixed model repeated measures ANOVA (within factor, area, and hemisphere; between factor, sex) at a significance level of α = 0.05.

## 3 Results

**Figure 3:**
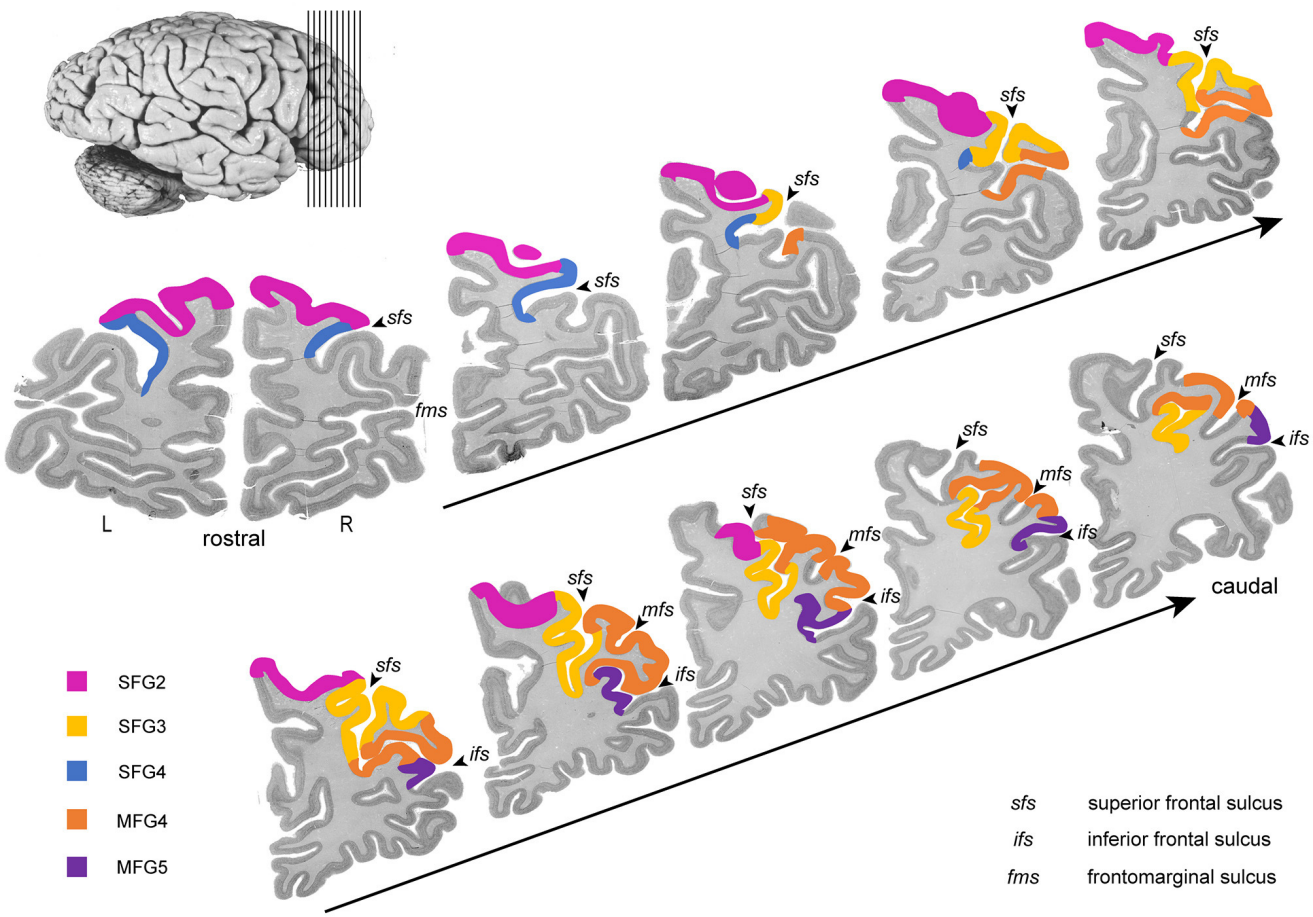
Rostro-caudal series of ten coronal sections through the right hemisphere. The new areas of DLPFC are highlighted in different colors (SFG2 in pink, SFG3 in blue, SFG4 in yellow, MFG4 in orange, and MFG5 in purple). Ten consecutive sections of the right hemisphere from anterior to posterior show the localization and the extent of the delineated areas regarding the macroscopical properties.

**Figure 4:**
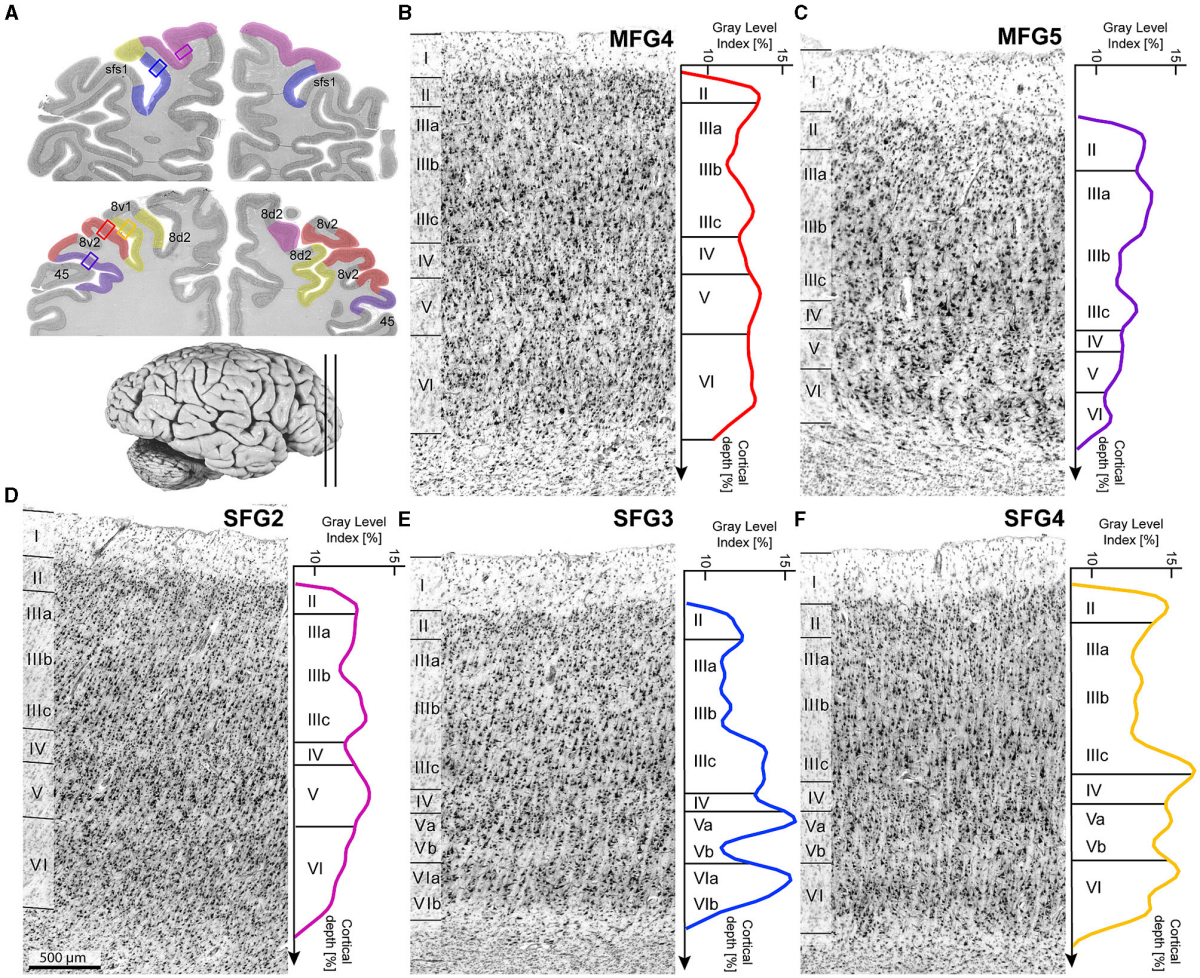
Cytoarchitecture and corresponding mean GLI-profiles. Selected slices **(A)** indicated area localizations and positions of the single cytoarchitectonic tiles. Colored lines with corresponding colors (see Figure 3) indicate mean GLI-profiles. GLI-profiles reflect the laminar changes in the volume fraction of cell bodies. SFG2 was characterized by large pyramidal cells in layer V **(D)**. Layer IV was poorly developed in area SFG3 **(E)** compared to area SFG4. Cells in upper layer IIIa were more loosely packed in area SFG4 **(F)** than in area MFG4 **(B)**. MFG5 was characterized by a sharp border to the white matter and dense layers II and V **(C)**. Histological sections were contrast-enhanced for better visualization. Scale bar with 500 μm in **(D)** refers to all **(B–F)**.

Five new cytoarchitectonic areas were identified within the DLPFC (Figures 3, 4): areas SFG2 (superior frontal gyrus 2), SFG3 (superior frontal gyrus 3), SFG4 (superior frontal gyrus 4), MFG4 (middle frontal gyrus 4), and MFG5 (middle frontal gyrus 5) according to their approximate macroanatomical location (see Figures 3, 4A) within the sulci and gyri (*sfg* and *mfg)*.

### 3.1 Cytoarchitectonic characteristics and borders

All areas SFG2, SFG3, SFG4, MFG4, and MFG5 showed six cortical layers, including layer IV, and, thus, represent a typical granular isocortex. However, individual and neighboring areas could be clearly separated based on their distinct cytoarchitectonic characteristics, such as size, density, and arrangement of neurons within single cortical layers (summarized in Table 2).

**Table 2:** Cytoarchitectonic characteristics of DLPFC areas SFG2, SFG3, SFG4, MFG4, MFG5 and neighboring areas.

**Area**		**Cytoarchitectonic characteristics**
SFG2	II III IV V VI	Cell dense and thick with a blurry border to layer III Subdividable with more loosely packed cells in layers IIIa and IIIb IIIc with medium-sized pyramidal cells Definable, but fewer cells compared to SFG4, MFG4, and MFG5 Medium to large-sized pyramidal cells, larger cells compared to IIIc, without subdivision Prominent and broad Blurry transition to white matter
SFG3	II III IV V VI	Thin with a clear border to layer III compared to SFG2 Pyramidal cell gradient along layer III with medium-sized pyramidal cells in deeper IIIc Poorly developed with blurry layers Sub-dividable with large pyramidal cells in Va and pale Vb with low cell density Cell dense with medium-sized pyramidal cells in VIa and thin pale VIb Sharp border to white matter
SFG4	II III IV V VI	Thin with a sharp border to layer III More loosely packed medium-sized cells in IIIa and IIIb compared to MFG4 and larger pyramidal neurons in IIIc Densely packed and well-definable Pyramidal neurons in Va are smaller compared to layer IIIc and pale layer Vb Cell dense Blurry white-matter border compared to SFG3
MFG4	II III IV V VI	Prominent but thin with a blurry border to layer III More cell dense compared to SFG4 with medium to large-sized neurons in deeper IIIc Well developed Medium to large-sized cells distributed across the layer Prominent, cell dense, and broad White-matter transition sharper as compared to SFG4
MFG5	II III IV V VI	Thin with a blurry transition to layer III High cell density with a gradient in cell size to IIIc Visible but thinner than in MFG4 Medium to large-sized pyramidal cells Thin and cell-dense Sharp border to white matter
Fp1		Sharp border between layers I, II, and III Dense layer II and deeper layer III Layer IV more pronounced than in SFG3 Pyramidal cells in layer V are not as large as in SFG2
SFS1		Homogenous appearance with uniformly packed layer III than SFG2 Dense, well-definable layer IV Medium-sized pyramidal cells in layer V and thin layer VI compared to SFG2 Sharp border between layer VI and white matter
SFS2		Sparser cell packing than in MFG4 Blurry, not well-definable layer IV Layer V and VI are less developed compared to MFG4
MFG1		Large pyramidal cells in deeper layer IIIc Broader layer IV than in SFS2 but not as dense as compared to MFG2 Prominent layer VI with large cells and blurry border to white matter
45		Large pyramidal cells in deep layer IIIc compared to MFG5 Layer IV visible but less developed than in MFG5 White matter transition bot as sharp as in MFG5
6d3		Agranular cortex with poor lamination across layers Pyramidal cell clusters in layer V
8d1		Cell-rich layer II compared to cell-poor superficial layer III with more prominent layer IIIc Layer IV as weak stripe, cell-rich superficial layer (Va), and stripe-like cell-poor layer Vb
8d2		Less densely packed granular layer II compared to 8d1 Thin, visible layer IV and large pyramidal cells in Va and a light band, poor in cells (Vb) High cell density in layer VI with a sharp contrast to white matter
8v1		Columnar arrangement of pyramidal cells in layers III to V with hardly prominent but Visible layer IV Very prominent layer VI with high volume and cell density
8v2		Narrow and densely packed layer II Highly distinctive stripe as layer IV and subdividable layer V Tightly packed cell layer in layer VI
ifs1		Subdivision of layer III with small densely packed neurons in deeper layer III Thin and narrow layer IV Indistinct border between layers V and VI
ifs3		Cells arranged in columns Indistinct layer IV Large pyramidal cells in deeper layer III

#### 3.1.1 Cytoarchitecture of areas SFG1, SFG2, SFG4, MFG4, and MFG5

In detail, area SFG2 had a prominent and cell-dense layer II with a blurred boundary to layer III, which could be subdivided due to increasing cell body size along the layer. Pyramidal cells in the deeper layer IIIc were medium-sized but smaller than neurons in layer V. Layer IV was very thin compared to other areas of the DLPFC (Figure 4B). The cell-rich and broad layer VI showed a blurry transition to the white matter in contrast to area SFG3.

Area SFG3 showed a thin layer II with a clear border to layer III. There was a slight gradient in pyramidal cell size in layer III from upper to lower parts. Layer IV was even more poorly demarcated than in area SFG2. Infragranular layers V and VI in area SFG3 could be subdivided based on their cell arrangement. Cells lay concentrated in the upper layers Va (local maxima in the GLI profile, see Figure 4C) and VI compared to the more cell-poor layers Vb (local minimum in the GLI profile) and VIb, which appeared pale. The white-matter boundary was sharply contrasted with the adjacent areas SFG2 and SFG4.

**Figure 5:**
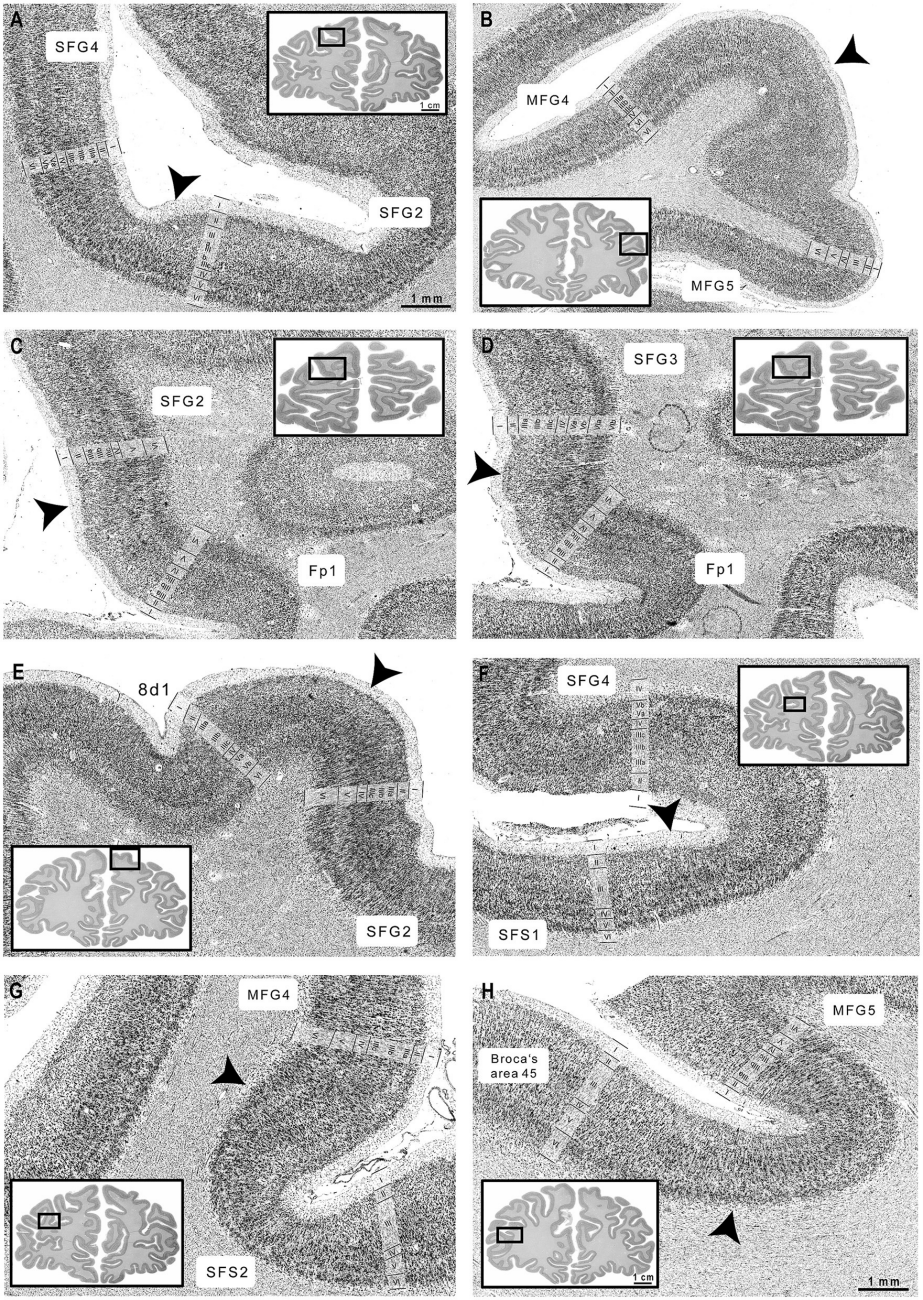
Cortical borders and cytoarchitecture of newly identified DLPFC areas. **(A)** SFG2 showed a broad layer II, while SFG4 revealed a thin layer II. **(B)** MFG4 showed a broad layer IV compared to area MFG5. **(C)** The characteristic feature of area SFG2 were blurry transitions to layer III, while Fp1 revealed sharp borders between layers I, II, and III. **(D)** SFG3 showed a well-definable layer IV and a visible border to the white matter compared to Fp1 (Bludau et al., 2014). **(E)** Area SFG2 could be clearly separated by the adjacent area 8d1 by the large pyramidal cells in layers IIIc and V. **(F)** SFG4 was characterized by a slight cell gradient across layer III, compared to the uniformly packed layer III of SFS1. **(G)** MFG4 showed dense, cell-rich layers IIIc and V, while SFS2 was less cell-rich. **(H)** Compared to area MFG5, Broca's area 45 showed large pyramidal cells in deeper layer III and blurry white-matter-boundary (Amunts et al., 1999). Scales of zoomed histological images: 1 mm. Scale of whole brain sections: 1 cm. Arrowheads indicate the cortical borders. Images were contrast-enhanced for better visualization.

The main characteristic feature of area SFG4 was the cell-dense and well-definable layer IV compared to the adjacent areas SFG2 (Figure 5A) and SFG3. The thin layer II showed a sharp transition to layer III with more loosely packed cells in IIIa and IIIb compared to area MFG4. The pyramidal cells in the deeper layer IIIc were larger than in upper Va. Cells in layer VI were densely packed with a blurred border to the white matter. The supragranular layers of the area MFG4 were broad and prominent. The thin layer II could not be clearly separated from layer III, as cells in upper layer IIIa were more densely-packed compared to the neighboring area SFG4. Layer IV was distinct, and layer V comprised medium to large cells mainly located in the upper layer Va (see local maximum in GLI profile, Figure 4D). Compared to area SFG4, the white-matter border was sharper.

Area MFG4 was mainly characterized by densely arranged cells, especially in the supragranular layers and layer IV. Layer II, in contrast to area SFG4, transitioned indistinctly into the cell-rich layer IIIa. There was a gradient of pyramidal cell size along layer III with medium to large neurons in deeper layer IIIc (local maximum in GLI profile, see Figure 4E). Layer IV was well-developed and could be clearly defined. The medium to large pyramidal cells in layer V showed no sublamination, and the prominent and broad layer VI displayed a sharper white-matter-border than area SFG4.

Ventrally to area MFG4 and dorsally to area 45 of the Broca's region (Amunts et al., 1999), area MFG5 was located mainly on the *mfg* and in the *ifs*. It showed a thin layer II without a sharp border to the cell-dense layer III, which displayed a gradient in cell size. Layer IV was visible but less definable compared to MFG4 (Figure 5B). Layer V, with its medium-sized neurons, could not be divided into Va and Vb, as reflected by the flat curve in the GLI profile (Figure 4F). The thin layer VI showed a high cell density and a sharp white matter border.

#### 3.1.2 Boundaries to neighboring areas and their cytoarchitectonic characteristics

The cytoarchitecture of SFG2, SFG3, SFG4, MFG4, and MFG5 differed from neighboring areas (see Table 2 and Figure 5). Ventrorostral to SFG2 and SFG3, the frontopolar area 1 (Fp1) (Bludau et al., 2014) was located, which could be distinguished from adjacent areas of the DLPFC based on specific cytoarchitectonic features (Figure 5C). Compared to area SFG2, Fp1 had a dense layer II with a sharper border to layer III, and layer IV was more prominent and cell-dense than in area SFG3. The pyramidal cells in layer V of area Fp1 were not as large as in area SFG2, and the white-matter-boundary was distinct compared to area SFG3 (Figure 5D).

In their caudal process, the newly delineated areas were displaced by the initial parts of the subdivisions of area 8. Area 8d1 appeared on the *sfg* and could be clearly distinguished by the poorly developed layer IV compared to adjacent areas SFG2 (Figure 5E) and SFG4. Area 8d2 showed less densely packed granular layers II and IV and a sharper border toward the white matter than area SFG2. Compared to the more ventrally located areas SFG4 and MFG4, layer IV of area 8d2 was thin and less defined. The pyramidal cells in layer V were larger, and the white matter boundary was sharper in area 8d2 than in area SFG4. Area 8v1 displayed a columnar arrangement of pyramidal cells in layers III and V and a very prominent layer VI with high volume and cell density in contrast to areas SFG4 and MFG4.

In the anterior parts of the DLPFC, newly delineated areas share borders with previously mapped areas, like SFS1 and SFS2 (Bruno et al., 2022). Compared to area SFG4, area SFS1 showed a homogenous appearance as the layers were composed of equally sized cells without larger pyramidal neurons in layers III or V (Figure 5F). Furthermore, layer IV was more cell-rich and well-developed in the area SFS1 than in the area SFG4. Whereas, layer IV was poorly developed in area SFS2 compared to MFG4 (Figure 5G).

Posteroventrally, area MFG4 shares borders with the areas ifs1 and ifs3 (Ruland et al., 2022), which are located within the *ifs*. Furthermore, area MFG5 borders posteriorly to area ifs1. Compared to the DLPFC areas, the ifs areas can be assigned to the dysgranular cortex since layer IV was thin and barely delineable. Whereas area ifs1 can be distinguished from ifs3 by loosely packed small pyramidal cells in the deeper layer III and a blurred V/VI boundary (Ruland et al., 2022).

Anteroventral to MFG5, the yet unpublished area MFG3, spanning the ascending aspect of the *ifs*, was identified, which seems to correspond to parts of BA46 based on preliminary mapping work. Compared to MFG5, this area shows distinct layers with small cells in the upper layer IIIa and a few large pyramidal cells in the deeper layer IIIc. Area MFG3 also showed a thin layer II with an indistinct border to layer III and a broad, well-developed layer IV. Layer V comprised medium-sized pyramidal cells in the upper layer Va and a pale Vb. Cells in layer VI were not as large, and the white matter border was less sharp than in area MFG5.

Posteroventrally to area MFG5, area 45 of Broca's region (Amunts et al., 1999) was located on the surface of the *ifg*. The main characteristic features of area 45 are large pyramidal cells in the deeper layer IIIc, a less pronounced layer IV and a blurred transition into the white matter compared to MFG5 (Figure 5H).

#### 3.1.3 Quantification of cytoarchitectonic differences and similarities of new areas and neighboring areas of the prefrontal cortex

**Figure 6:**
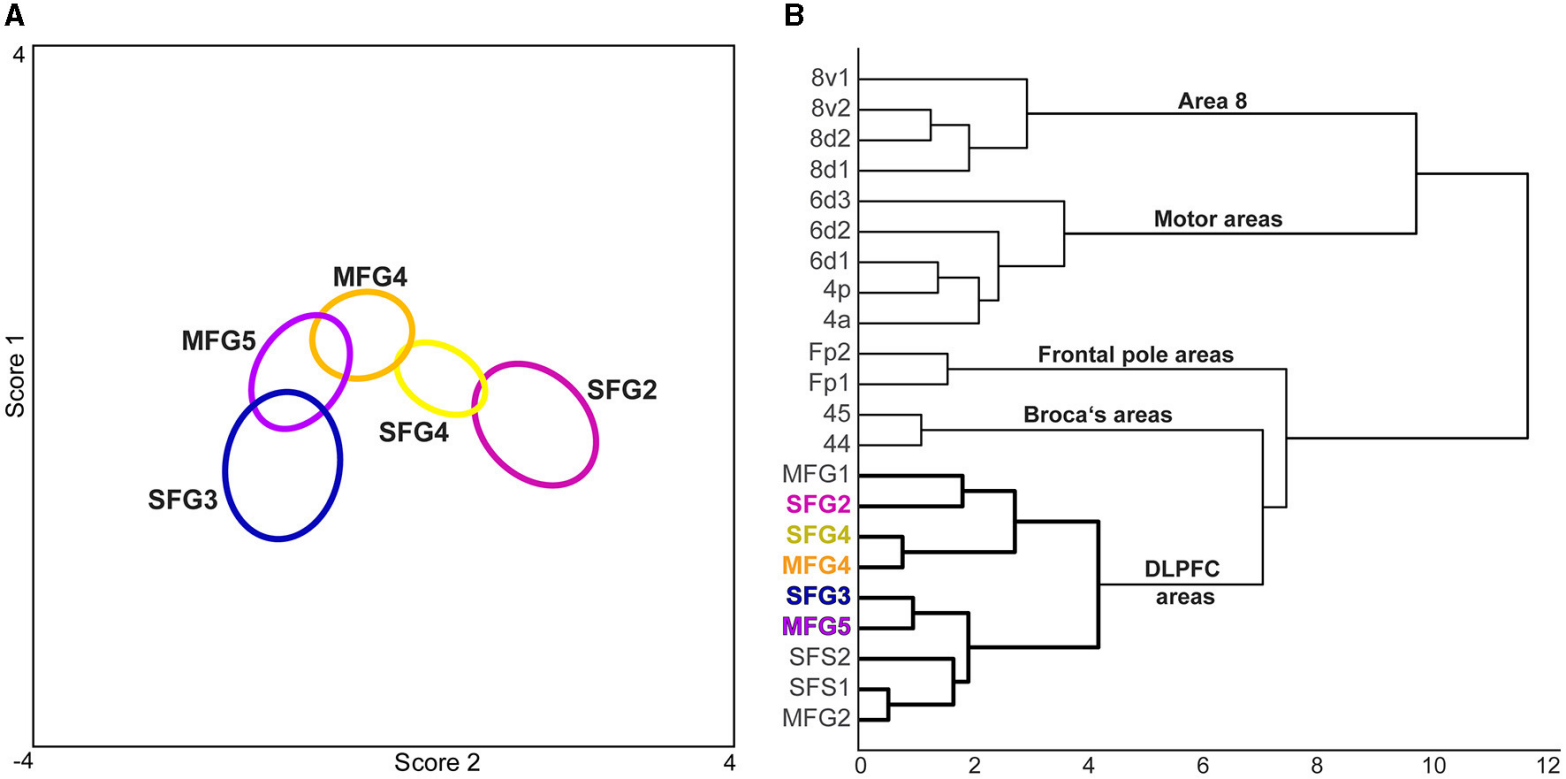
Discriminant and cluster analysis of the five new DLPFC areas and adjacent regions. **(A)** In the discriminant analysis, the cytoarchitecture of each newly identified area was distinguishable and could be separated due to distinct features. The centroids (ellipsoids) of the identified areas comprise the gray level index (GLI) profiles of the individual brains of each area. **(B)** The GLI profiles were compared with neighboring areas of the frontal lobe in a cluster analysis. Areas of the motoric region [4p, 4a (Geyer et al., 1996), 6d1, 6d2 and 6d3 (Amunts et al., 2021)] and subdivisions of area 8 [8d1, 8d2, 8v1, 8v2 (Amunts et al., 2021)] showed a high Euclidean distance indicating high cytoarchitectonic dissimilarity. The dendrogram shows a grouping of Broca's areas [44, 45 (Amunts et al., 1999) and frontal pole areas Fp1, Fp2 (Bludau et al., 2014)] vs. the newly identified areas, which clustered with anterior DLPFC areas [SFS1, SFS2, MFG1, MFG2 (Bruno et al., 2022)].

A hierarchical cluster analysis based on GLI profiles demonstrated a higher cytoarchitectonic similarity within the DLPFC areas compared to neighboring areas of the frontal pole Fp1 and Fp2 (Bludau et al., 2014) and Broca's areas 44 and 45 (Amunts et al., 1999). Subdivisions of area 8 [8d1, 8d2, 8v1, and 8v2 (Amunts et al., 2021], and motoric areas [4p, 4a (Geyer et al., 1996), 6d1, 6d2 and 6d3 (Amunts et al., 2021)], were clearly separable to DLPFC areas. Compared to the more distant areas of the frontal lobe (frontal pole areas, VLPFC areas, and motoric areas), the formerly published areas of the anterior DLPFC (Bruno et al., 2022) show a high cytoarchitectonic similarity with the newly identified DLPFC areas (Figure 6B). Area SFG2 clustered with the area MFG1, an anterior DLPFC area (Bruno et al., 2022). SFG3 formed a cluster with a low Euclidean distance to MFG5 while separated from the other newly identified areas. The two areas have a thin layer IV compared to adjacent areas. At the same time, these two areas are rather distant from each other regarding their topography. In contrast to that, areas MFG1 and MFG2 are located rather closely to each other while their cytoarchitecture is different as indicated in Figure 6. SFG4 clustered with the adjacent DLPFC area MFG4 as these areas share distinct cytoarchitectonic characteristics like medium to large-sized neurons in layer IIIc and a well-developed layer IV.

### 3.2 Interindividual variability

#### 3.2.1 Individual localization of areas within the single brains

**Figure 7:**
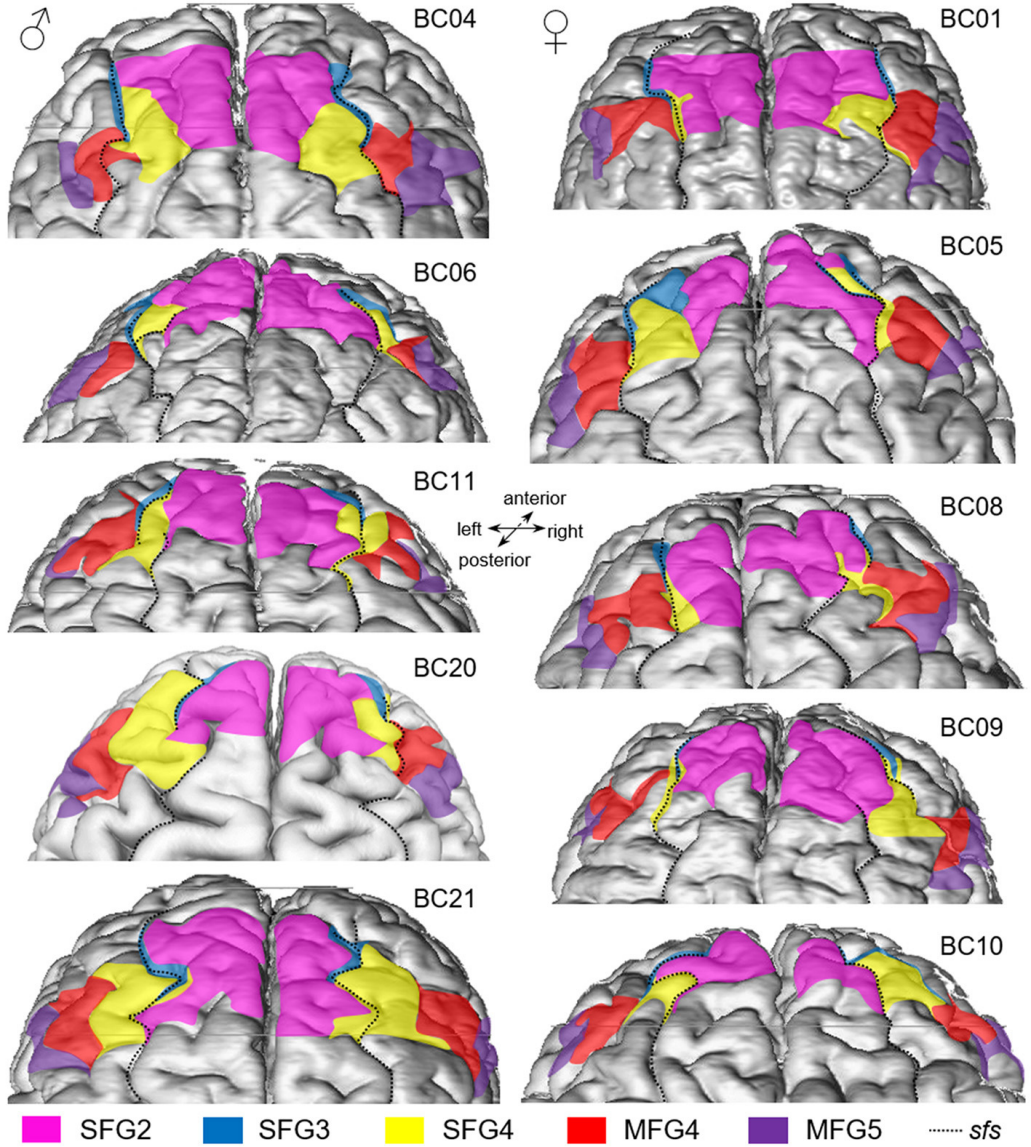
Dorsal view of 5 female **(left column)** and 5 male **(right column)** 3D-reconstructed brains. Areas SFG2 (pink), SFG3 (blue), SFG4 (yellow), MFG4 (red), and MFG5 (purple) vary between the different in size and shape and show a varying sulcal pattern of the DLPFC. The dotted black line indicates the course of the superior frontal sulcus (*sfs*).

Examination of area extent and localization revealed that the cytoarchitectonically delineated boundaries between areas did not compulsory correspond to the sulcal and gyral landmarks. Dorsal to the frontal pole area Fp1 (Bludau et al., 2014), area SFG2 covered most of the surface of the *sfg* but also partially extended to the descending and ascending banks of the *sfs*. Area SFG3 was located ventrally to area SFG2, mainly on the ascending ventral bank of the *sfs* but partly reaching the surface of the *sfg* (see Figure 7 BC05 left hemisphere). In its caudal process, area SFG3 was displaced by the emerging area SFG4, which could be identified within the *sfs*, on the *sfg*, and the *mfg*. In detail, in eight out of 20 hemispheres analyzed, area SFG4 mainly spanned over the surface of the *sfg* (see, for example, Figure 7 BC04). In six hemispheres, the area was mainly located on the surface of the *mfg* (see Figure 7 BC 21). In the four hemispheres, it was mainly located within the depth of the *sfs* (see, for example, the right hemisphere of BC08 in Figure 7). On the more ventral parts of the *mfg*, area MFG4 was situated. MFG4 extended into the middle frontal sulcus (*mfs)* where present. A *mfs* was present in 17 out of the 20 hemispheres examined. Adjacent to area MFG4, area MFG5 was located in the ascending and descending part of the *ifs* but also reached the top of the *mfg* and *ifg*, bordering area 45 of Broca's region (Amunts et al., 1999).

#### 3.2.2 Probability maps and maximum probability maps

**Figure 8:**
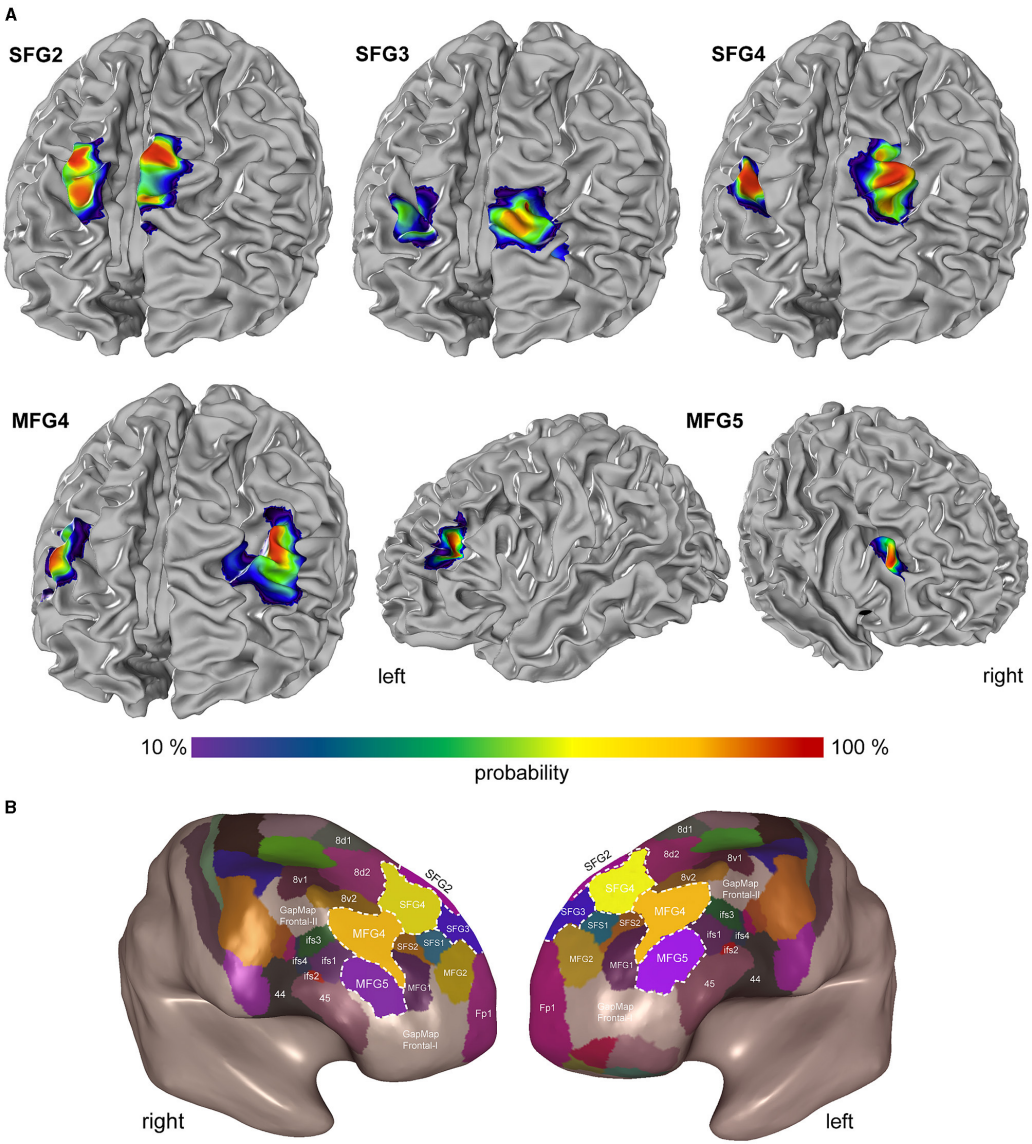
Probability maps and Maximum Probability Map in the DLPFC. **(A)** The probability maps are shown on the individual anatomical template brain MNI Colin27. The color-coded probability maps represent interindividual variability. Probability values range from 10% (violet-blue) to 100 % (red). **(B)** Maximum probability maps as the inflated view of the MNI Colin27 in smooth-white-matter mode demonstrate area localization on gyri and sulci. This non-overlapping surface illustrates the position of SFG2 (pink), SFG3 (blue), SFG4 (yellow), MFG4 (red), and MFG5 (purple) with the neighboring frontal pole area Fp1 (magenta; Bludau et al., 2014), subdivisions of area 8 (8d1, 8d2, 8v1, and 8v2), areas of Broca's region [44 and 45 (Amunts et al., 1999)] and yet umapped regions (GapMap Frontal-I and GapMap Frontal-II).

Probability maps of the areas (SFG2, SFG3, SFG4, MFG4, and MFG5) were computed in the two stereotaxic spaces MNI Colin27 and ICBM152casym (Figure 8) to quantify the intersubject variability in the extent and location of the DLPFC. The probability maps show the overlap of areas with a color gradient from red (high probability and low intersubject variability) to blue (low probability and high intersubject variability). For both reference spaces, the centers of gravity of the new DLPFC areas are shown in Table 3.

**Table 3:** Center of gravity coordinates in MNI Colin27 and ICBM152casym space of new areas and attributed to Yeo 17 networks.

**Area**		**ICBM152casym**			**MNI Colin27**			**Yeo 17 networks**
		***x***	***y***	***z***	***x***	***y***	***z***	
SFG2	Left Right	−11 15	52 49	37 38	−9 16	51 48	41 41	DefaultB DefaultA/B
SFG3	Left Right	−16 18	57 57	21 21	−18 22	53 52	24 27	DefaultB DefaultB
SFG4	Left Right	−25 26	42 46	32 27	−22 28	43 41	35 36	SalVentAttnB SalVentAttnB
MFG4	Left Right	−37 35	44 45	26 24	−35 37	40 38	30 31	SalVentAttnB/ContA SalVentAttnB
MFG5	Left Right	−40 40	41 43	16 15	−40 46	38 33	20 24	ContA ContA
**Anterior DLPFC areas (Bruno et al.**, **2022****)**								
SFS1	Left Right	−27 25	50 52	21 21	−25 26	48 50	22 20	SalVentAttnB SalVentAttnB
SFS2	Left Right	−31 28	47 50	25 23	−29 30	46 48	27 22	SalVentAttnB SalVentAttnB
MFG1	Left Right	−38 37	50 50	20 21	−36 39	50 48	23 22	SalVentAttnB SalVentAttnB
MFG2	Left Right	−26 30	53 55	17 11	−25 31	52 52	18 11	SalVentAttnB/ContB SalVentAttnB/ContB

SFG2 is located exclusively on the rostral *sfg*, starting from the superior border of the cerebral hemisphere and extending to the *sfs*. In this sulcus, SFG3 is located mainly on the rostral side and extends to the fundus of the *sfs*. SFG4 was situated in the ascending part of the *mfg*. With decreasing probability, SFG4 also develops at the gyral border to the *sfs*. MFG4 also expands on the *mfg*, primarily on the surface, less likely in the rostral area of the *sfs*, extending into the *mfs*. MFG5 extends over the *mfg* into the *ifs* (Figure 8A).

A non-overlapping surface representation of all five DLPFC regions is provided by the MPM, showing the topography of the areas and the cytoarchitecturally defined adjacent region Fp1 (Bludau et al., 2014), the anterior DLPFC areas (SFS1, SFS2, MFG1, MFG2), areas 8d1, 8d2, 8v1, and 8v2, and Broca's areas 44 and 45 (Amunts et al., 1999) of the ventral prefrontal cortex on the inflated brain surface of MNI Colin27 (Figure 8B). The newly identified areas are located in the “GapMap Frontal-I” brain region, an unmapped region in the frontal lobe region (Amunts et al., 2020).

The new maps are publicly available and free to share under the Creative Commons license agreement and can be downloaded (ebrains.eu). They are part of the Julich Brain Atlas and can be analyzed using the Interactive Atlas Viewer of the siibra explorer (atlases.ebrains.eu/viewer).

### 3.3 Volumes of areas SFG2, SFG3, SFG4, MFG4, and MFG5

Differences in the shrinkage-corrected volumes of the five areas showed that area SFG2 had the largest volume (5,711 ± 324 mm^3^), followed by SFG4 (4,281 ± 226 mm^3^), MFG4 (4,031 ± 167 mm^3^), MFG5 (3,630 ± 112 mm^3^), and SFG3 (1,504 ± 132 mm^3^). The combined cortical volume of DLPFC areas was 10,029 ± 239 mm^3^ in the right hemisphere and 9,128 ± 267 mm^3^ in the left hemisphere. Male brains had a total volume of 22,981 ± 785 mm^3^, with 11,648 ± 397 mm^3^ in the right hemisphere and 11,333 ± 388 mm^3^ in the left hemisphere. Female brains had a total volume of 15,334 ± 444 mm^3^, with 8,410 ± 248 mm^3^ in the right and 6,924 ± 196 mm^3^ in the left hemisphere.

Differences in the shrinkage-corrected volumes of the five areas were analyzed for interhemispheric and sex differences. Shrinkage-corrected area volumes were normalized to the corresponding total brain volume and then compared using an ANOVA (within factors, area and hemisphere; between factor, sex). No significant volume differences between sexes and hemispheres were found.

## 4 Discussion

Extending our previous research of mapping the frontal lobe, this study adds five new cytoarchitectonic areas (SFG2, SFG3, SFG4, MFG4, and MFG5). They contribute to the Julich Brain Atlas, leaving only a few uncharted spots. An imaging analysis approach based on GLI-profiles enabled reliable and statistically reproducible testing of areal boundaries and characteristics. The interindividual variability concerning area extent and relationship to macroanatomical properties was captured in 3D probability maps in two common reference spaces. These maps allowed the comparison with activations obtained from functional neuroimaging studies to further unravel this brain region's functional parcellation.

### 4.1 Microstructural organization of the DLPFC

The human DLPFC was microstructurally parcellated into distinct areas by former researchers (Brodmann, 1909; Rajkowska and Goldman-Rakic, 1995a; Petrides and Pandya, 1999). However, there is evidence that former maps do not adequately reflect the whole functional diversity of this brain region as described by functional parcellations (Yeo et al., 2011; Shen et al., 2013; Schaefer et al., 2018). As it has already been shown by Bruno et al. (2022) that parts of the anterior DLPFC can be subdivided into four subregions, we now focused on additional adjacent areas using the same methodological approach (Schleicher et al., 1999; Amunts et al., 2020) at the microstructural level. We were able to delineate a further five areas based on their cytoarchitecture.

In contrast to former microstructural maps, the present study provides three-dimensional probability maps in reference spaces with a finer subdivision of the human DLPFC based on reliable observer-independent image analysis. As the cytoarchitectonic parcellation of Petrides and Pandya (1999) is the most recent map of the DLPFC and shows the highest unity with our results concerning subdivisions, we are focusing on that study for comparison. They subdivided the DLPFC into four distinct regions. Area 9 covers the surface of the *sfg* but also extends medially to the paracingulate sulcus. The main cytoarchitectonic features were compact layers II and IIIa, large pyramidal cells in the deeper layer IIIc and a thin, poorly developed layer IV. Area 46 has a uniform appearance with medium-sized pyramidal cells in layers IIIc and Va and a broad cell-dense layer IV. The area was mainly in the *mfs* and the middle parts of the *mfg*. The rest of the *mfg* was covered by area 9/46, which could be divided into a dorsal and ventral part. Area 9/46 has a well-developed layer II with a distinct boundary to layer III. Furthermore, there are considerably large pyramidal cells in the deeper layer IIIc, a well-developed layer IV, medium-sized cells in layer Va and a sparse layer Vb. In area 9/46d, the pyramidal cells in layer IIIc are less densely packed, and layer IV is thinner than in the ventral part 9/46v.

Compared to the areas delineated in the present study, the cytoarchitectonic descriptions of Petrides and Pandya (1999) fit well, although not entirely. Area SFG2 shows the same characteristics as area 9, but the extent of area SFG2 is limited to the dorsal aspect of the frontal lobe and does not extend to the mesial surface compared to area 9. Area SFG3 has a poorly developed layer IV like area 9 but shows some difference in infragranular layers, such as separable layers V and VI. The microstructural features of 9/46d, like a distinct transition of layer II to III, large pyramidal neurons in deeper layer IIIc, and a well-definable layer IV, can be assigned to the area SFG4. Furthermore, layer V consists of medium-sized pyramidal cells in layer Va and sparse layer Vb. Area 9/46v covers the more ventral parts of the *mfg* in the former map. However, the newly delineated area SFG4 extends to the *sfg* and partly to the *mfg* and *mfs* and thus does not coincide entirely with the described location of the area 9/46d, which was mainly located on the *mfg*.

Comparing the area localization of MFG4 on the *mfg* with the former map of Petrides and Pandya (1999), MFG4 overlaps with the area 9/46v. Both areas have a broader and more pronounced layer IV compared to adjacent areas SFG4 or 9/46d, respectively. However, the boundary between layers II and III was not as distinct, and we could not detect a sparse layer Vb in area MFG4 compared to area 9/46v. Area MFG5 shares some cytoarchitectonic characteristics of the formerly described area 9, like a thin layer IV. However, there were also microstructural differences. For example, layer V was not composed of large pyramidal cells, but neurons in deeper layer IIIc were larger than layer V neurons.

Relating these comparisons with the result of the performed cluster analysis and the previously published data (Bruno et al., 2022), the DLPFC can be described with respect to its cytoarchitectonic organization as follows: Areas MFG1, SFG2, SFG3, SFS2, and MFG5 reveal main characteristics of area 9 as described in former microstructural maps (Brodmann, 1909; Rajkowska and Goldman-Rakic, 1995a; Petrides and Pandya, 1999), like a thin layer IV and large pyramidal cells in the pyramidal layers. However, these areas differ from each other, as shown by image analysis statistical tests, and cannot be merged into a single area. The present parcellation shows a more fine-grained parcellation than previous maps (Brodmann, 1909; Rajkowska and Goldman-Rakic, 1995a; Petrides and Pandya, 1999). Areas SFS1 and MFG2, with their homogeneous appearance and well-developed layers IV, could correspond to area 46 as defined by Rajkowska and Goldman-Rakic (1995a) as well as Petrides and Pandya (1999). Areas SFG4 and MFG4, clustering together, seem to resemble the transition area 9/46 (Rajkowska and Goldman-Rakic, 1995a; Petrides and Pandya, 1999). But they share more cytoarchitectonic similarity with areas MFG1 and SFG2 than to the remaining DLPFC areas (see Figure 6).

The cluster analysis revealed no correlation between cytoarchitectural similarity and topography. In general, DLPFC areas of the *sfg* clustered with areas on the *mfg*: For example, SFG2 clustered with MFG1, and SFG4 with MFG4. This result indicates that no clear gradient could be observed along a topographical axis. It is reasonable to assume that the DLPFC has an internal functional organization, and that correlations are more likely to be found on a structural-functional level, as discussed in the following section.

### 4.2 Structural-functional properties of the human DLPFC

Up to now, nine microstructurally distinct areas within the human DLPFC could be delineated based on the cytoarchitecture, including the newly identified areas and a previous cytoarchitectonic study (Bruno et al., 2022). This new fine-resolved map now allows us to locate functions more precisely from activation studies. A first comparison of the areas with neuroimaging data on brain function suggests distinct contributions that will be discussed with existing functional studies and parcellations (Yeo et al., 2011; Glasser et al., 2016) in the following sections.

Yeo et al. (2011) studied the organization of the human cerebral cortex based on intrinsic functional connectivity, resulting in different functional networks. The DLPFC coordinates of the present study were compared to the study of Yeo et al. (2011) by their center of gravity MNI coordinates (Table 3). This analysis revealed that SFG2 and SFG3 can be associated with the default mode network. SFG4 and MFG4 of both hemispheres could be associated with the salience/ventral attention network, while MFG5 is part of the frontoparietal control network (ControlA).

Several studies have shown that SFG2 is functionally involved in self-reflection, e.g., the area is the location of a common activation at rest and during the self-referential task (D'Argembeau et al., 2005), self-evaluation (Ochsner et al., 2005), self-representation (Debbane et al., 2017), and cognitive empathy (Schnell et al., 2011). The brain's default mode network has become closely associated with self-referential mental activity, particularly in the resting state (Davey et al., 2016). Based on the visual assignment in the functional atlas of Yeo and colleagues, SFG2 can be assigned to the default network (Yeo et al., 2011). When new information is introduced, the ventral attention network shifts the attention (Vossel et al., 2014). SFG2 clusters with MFG1 (Bruno et al., 2022, Figure 6B), which can be assigned to this attentional network.

Little is known about the functional role of the region where area SFG3 is located. One imaging study has shown that the area is active in fragment-based retrieval in the right DLPFC and thus plays a role in episodic retrieval (Allan et al., 2000). The area has also been shown to be active in episodic memory (Smith et al., 2007). Episodic memory retrieval processes with strong memory content recruit the default network (Kim, 2016), and the successful retrieval from episodic memory involves the frontoparietal network (Iidaka et al., 2006). Thus, the results of the imaging studies are consistent with the network analysis (Yeo et al., 2011), that SFG3 could be assigned to the default network. SFG3 clusters with MFG5, an area that could be allocated to the frontoparietal control network (ControlA, Figure 6).

The coordinates for SFG4 seem to be implicated in the salience/ventral attention network (Yeo et al., 2011). The salience network is essential in tasks involving attention and responding to unexpected but salient stimuli (Sridharan et al., 2008; Menon and Uddin, 2010). The bottom-up attentional process involves the ventral attentional network (Vossel et al., 2014). Imaging studies have shown activation of the area in error awareness (Masina et al., 2018) and self-control (Hayashi et al., 2013). SFG4 clusters with MFG4 according to cytoarchitectonic similarities. However, like SFG4, the coordinates of the probabilistic map of MFG4 seem to be part of the salience/ventral attention network (Yeo et al., 2011). A previous study attributed MFG4 to working memory connected to the anterior cingulate cortex (Cieslik et al., 2013). Further studies found activations in the center of MFG4 during successful self-control (Harris et al., 2013; Chen et al., 2018; Ren et al., 2023) and self-regulation leading to better working memory performance (Zhang et al., 2013). Several studies consider the DLPFC as part of a large attentional network involving perceptual, attentional, and working memory (Yamasaki et al., 2002; Buschman and Miller, 2007; Zanto and Gazzaley, 2009; Lennert and Martinez-Trujillo, 2011). This broad functional profile of MFG4 can be summarized as attentional filtering to support self-control by reducing irrelevant stimuli (Zanto and Gazzaley, 2009; Harris et al., 2013).

MFG5 could be bilaterally assigned to the frontoparietal control network (Yeo et al., 2011), which is supported by other comparisons with functional activation studies. For example, DLPFC-mediated top-down control interferes with contextual memory-guided attention (Rosero Pahi et al., 2020), and MFG5 is active in the self-control of choice behavior (Schmidt et al., 2018). The frontoparietal network of the DLPFC is involved in cognitive control and working memory (Harding et al., 2015; Marek and Dosenbach, 2018). Indeed, MFG5 has also been shown to be involved in working memory processes, particularly in processing verbal input (Henson et al., 2000; Barbey et al., 2013).

In the multimodal parcellation of Glasser et al. (2016), the DLPFC is subdivided into 13 areas, including areas from the posterior part (8Ad, 8BL, 8Av and 8C) and premotor region (s6-8, i6-8, and SFL). Based on the results of the cluster analysis in this study (see Figure 6), which shows a clear separation of the posterior areas from the DLPFC areas, we suggest, like others (Jung et al., 2022), that these should not be included in the DLPFC by definition. Without these posterior areas, the DLPFC is comprised of the six areas 9a, 9p, 9-46d, 46, a9-46v, and p9-46v in the Glasser atlas (Glasser et al., 2016). Results from this and a previous study (Bruno et al., 2022) lead to a parcellation with so far nine areas (SFG2, SFG3, SFG4, SFS1, SFS2, MFG1, MFG2, MFG4, and MFG5). Based on a currently ongoing mapping study, we assume that the DLPFC can be microstructurally divided into at least 10 distinct areas.

Comparing the Glasser et al. (2016) map with our parcellation (see Figure 8), similarities as well as discrepancies can be found and will be discussed in the following. With its position on the most dorsal part of the *sfg* and borders to a subdivision of area 8, the area SFG2 matches the described area 9p. The same conditions are present for the areas SFG3 and 9a, which are adjacent to the frontal pole area (Fp1 or 10p) and located on the more anterior parts of the *sfg*. However, regarding the probability maps of our areas, area SFG3 is located to a large extent within the *sfs* and not on the surface of the *sfg*.

Area SFG4 shows similarities with the described area 9-46d, as it shares borders with the areas on the sfg (SFG2 and SFG3 or 9a and 9p, respectively). Regarding the entire area extension and the posterior borders, the areas SFG4 and 9-46d do not correspond. Area SFG4 borders posteriorly to the subdivisions of area 8, like area 8d2 and 8v2, whereas 9-46d mainly borders area 46 and only to a very small extent with area 8Ad (Glasser et al., 2016). This difference that area 46 borders posteriorly to areas 8Ad and 8Av (Glasser et al., 2016) is not given in our current map and cannot be found in any former cytoarchitectonic maps (Brodmann, 1909; von Economo and Koskinas, 1925; Sarkissov et al., 1955; Rajkowska and Goldman-Rakic, 1995b; Petrides and Pandya, 1999).

Area MFG4 can be related to the described area p9-46v (Glasser et al., 2016) concerning its location on the *mfg* dorsally to the *ifs* areas and anteriorly to the subdivisions of area 8. In contrast, the total area volume of MFG4 seems considerably larger compared to area p9-46v.

Area MFG5 shows similarities with the described area IFSa in its shape and location dorsal to Broca's area 45. However, area MFG5 was located to notable parts on the surface of the *mfg* and only to some extent in the *ifs* compared to the exclusively sulcal area IFSa (Glasser et al., 2016). Therefore, and in regard to the results of the cluster analysis (see Figure 6), we prefer the definition as MFG area and the assignment of the DLPFC instead of the VLPFC.

## 5 Conclusion

Based on the present and previous cytoarchitectonic studies (Bruno et al., 2022), the DLPFC cannot be seen as a singular and uniform region. Instead, it comprises distinct areas with unique cytoarchitectural characteristics that could be linked to different functional networks. The cluster analysis revealed that the areas could be assigned based on their cytoarchitecture and network affiliation. Overall, there are three superordinate networks: the frontoparietal network, the default network, and the salience/attentional network, to which all DLPFC areas investigated could be categorized. Thus, the cytoarchitectural analyses complement the functional studies (Yeo et al., 2011) and parcellations (Glasser et al., 2016) while providing a more precise area delineation within the DLPFC-associated regions. Finally, the five new cytoarchitecturally distinct areas (SFG2, SFG3, SFG4, MFG4, MFG5) within the DLPFC provide a new basis to gain a more comprehensive understanding of the role of the prefrontal cortex in cognitive control. Preliminary unpublished results from our research group show three more cytoarchitectonic areas in the DLPFC and three in the VLPFC. Once the analysis of all present areas in the DLPFC has been completed, detailed conclusions of the structural-functional organization of the DLPFC can be formulated.

## Data availability statement

The datasets presented in this study can be found in online repositories. The names of the repository/repositories and accession number(s) can be found in the article/supplementary material.

## Ethics statement

The used post-mortem tissue in this study was obtained through the body donor program of the medical faculty of the Heinrich-Heine-University Düsseldorf and approved by the Ethics Committee of the same institution (# 4863).

## Author contributions

AB: Data curation, Formal analysis, Investigation, Methodology, Validation, Visualization, Writing—original draft, Writing—review & editing. KL: Data curation, Formal analysis, Investigation, Methodology, Validation, Visualization, Writing—original draft, Writing—review & editing. SB: Methodology, Software, Visualization, Writing—review & editing. HM: Methodology, Software, Visualization, Writing—review & editing. KA: Conceptualization, Funding acquisition, Project administration, Resources, Supervision, Validation, Writing—review & editing.
